# Targeted next-generation sequencing of the 16S-23S rRNA region for culture-independent bacterial identification - increased discrimination of closely related species

**DOI:** 10.1038/s41598-017-03458-6

**Published:** 2017-06-13

**Authors:** Artur J. Sabat, Evert van Zanten, Viktoria Akkerboom, Guido Wisselink, Kees van Slochteren, Richard F. de Boer, Ron Hendrix, Alexander W. Friedrich, John W. A. Rossen, Anna M. D. (Mirjam) Kooistra-Smid

**Affiliations:** 1 Department of Medical Microbiology, University of Groningen, University Medical Center Groningen, Groningen, The Netherlands; 2 Department of Medical Microbiology, Certe, Groningen, The Netherlands

## Abstract

The aim of this study was to develop an easy-to-use culture-free diagnostic method based on next generation sequencing (NGS) of PCR amplification products encompassing whole 16S-23S rRNA region to improve the resolution of bacterial species identification. To determine the resolution of the new method 67 isolates were subjected to four identification methods: Sanger sequencing of the 16S rRNA gene; NGS of the 16S-23S rRNA region using MiSeq (Illumina) sequencer; Microflex MS (Bruker) and VITEK MS (bioMérieux). To evaluate the performance of this new method when applied directly on clinical samples, we conducted a proof of principle study with 60 urine samples from patients suspected of **urinary tract infections** (UTIs), 23 BacT/ALERT (bioMérieux) positive blood culture bottles and 21 clinical orthopedic samples. The resolution power of NGS of the 16S-23S rRNA region was superior to other tested identification methods. Furthermore, the new method correctly identified pathogens established as the cause of UTIs and blood stream infections with conventional culture. NGS of the 16S-23S rRNA region also showed increased detection of bacterial microorganisms in clinical samples from orthopedic patients. Therefore, we conclude that our method has the potential to increase diagnostic yield for detection of bacterial pathogenic species compared to current methods.

## Introduction

Timely, appropriate treatment of infection depends on rapid and specific identification of causative microorganisms. To date, identification of bacterial species highly depends on culture or molecular tests, including 16S rRNA gene Sanger sequencing^1, 2^. Significant limitations of culture methods are that some bacteria are slow-growing or fastidious, making identification of them complicated and time and resource consuming^3^. Although molecular tests that target specific microorganisms are more rapid and sensitive than culture methods they need an a priori knowledge of the likely pathogenic species that could be present in a sample. If bacteriological identification by culture methods fails, Sanger sequencing of the variable 16S rRNA gene is used for identification. The gene sequence has been proven to be a reliable genetic marker as it is present in all bacteria and its function has not changed over time^4^. However, unequivocal identification is not always possible due to the high sequence similarities of the 16S rRNA gene in some bacterial species^5^. Although Sanger sequencing of the 16S rRNA gene can be applied directly on clinical materials, in polymicrobial samples it usually cannot identify more than one species simultaneously or at least this process is challenging by sorting out the ambiguous signals from mixed chromatograms using a computer program^6^.

Next generation sequencing (NGS) allows culture free detection of a theoretically unlimited number of pathogens and thus provides insight in the full microbiome. Since the introduction of benchtop sequencers, NGS is likely to become a diagnostic tool within the next few years in microbiological laboratories^3, 7, 8^. Metagenomics will be the ultimate approach in detecting all microorganisms (e.g. bacteria, viruses, fungi) in a sample. Unfortunately, analysis of large datasets requires a combination of bioinformatics skills and computational resources that is nowadays mostly absent in diagnostic (medical) microbiological laboratories. Furthermore, metagenomics approaches are time consuming as the turnaround time is approximately 4–5 days. To fill the gap between the conventional methods (culture and PCR) and metagenomics, a culture free approach using targeted NGS will be an excellent approach to detect and identify bacterial species as, compared to metagenomics, it is less complicated, and cheaper and therefore more likely to get implemented in diagnostic laboratories within a short timeframe.

The aim of the present study was to develop a rapid and easy-to use culture free diagnostic method based on NGS of PCR amplification products encompassing the whole 16S-23S rRNA region to improve the resolution of bacterial species identification. Moreover, the new method was compared with three commonly used identification methods. Finally, the feasibility of the new identification method to detect and identify bacterial species in clinical specimens was evaluated.

## Results

### Comparison of identification potential of the tested methods

The results of bacterial identification obtained by the four tested methods are shown in Supplementary Table S1. The rates of accurate identification to the species level using NGS of the 16S-23S rRNA region, Sanger sequencing of the 16S rRNA gene, Microflex MS and Vitek MS methods were 92.5%, 56.7%, 73.1% and 64.2%, respectively. The rates of accurate identification to the genus level using NGS of the 16S-23S rRNA region, Sanger sequencing of the 16S rRNA gene, Microflex MS and Vitek MS methods were 100%, 94.0%, 88.1% and 83.6%, respectively. At the species and genus level, assessment of the statistical significance of the differences in accuracy of the four methods for assigning species and genus showed statistically significant differences between NGS of the 16S-23S rRNA region versus all other tested methods for all bacterial isolates (P < 0.05). The only exception was observed between the two sequencing methods at the genus level (P = 0.2482). Moreover, the Sanger sequencing of the 16S rRNA gene was significantly more discriminative at the genus level than Vitek MS (P < 0.05).

NGS of 16S-23S rRNA region was the only method which correctly identified to the species level *Shigella sonnei*, *Streptococcus oralis*, *Streptococcus mitis*, *Streptococcus vestibularis*, *Abiotrophia para-adiacens* and *Achromobacter marplatensis* (Table S1). Sanger sequencing of 16S rRNA gene was the only method which did not have a potential to identify *Corynebacterium ulcerans*, *Escherichia coli*, *Enterococcus faecium*, *Streptococcus parasanguinis*, *Staphylococcus aureus*, *Staphylococcus capitis*, *Staphylococcus epidermidis*, *Lactobacillus rhamnosus*, *Delftia acidovorans* and *Klebsiella pneumoniae*. Vitek MS showed identical and Bruker MS almost identical potential compared to NGS of the 16S-23S rRNA region for identification of the *Staphylococcus* isolates to the species level. Moreover, Bruker MS was the only method which correctly identified *B*. *cereus* and *L*. *paracasei* to the species level, and *Pseudomonas aeruginosa* was identified only by Vitek MS. Both Mass Spectrometry methods could not identify *Acinetobacter bereziniae*, *Corynebacterium afermentans*, *Corynebacterium pseudogenitalium*, *Salmonella enterica*, *Streptococcus tigurinus* and *Moraxella lacunata*. Moreover, Vitek MS could not identify *Acinetobacter parvus*, *Acinetobacter baumannii*, *Corynebacterium amycolatum*, *Corynebacterium coyleae*, *Streptococcus dysgalactiae* and *Actinomyces graevenitzii*, and Bruker MS had a problem with identification of *Staphylococcus lentus* to the species level. No method could unambiguously identify *S*. *pasteuri* to the species level (Table S1).

### Urine samples

Sixty fresh urine samples from patients suspected for urinary tract infections (UTIs) were subjected to conventional culture-based methods and culture-independent NGS of the 16S-23S rRNA region. Bacterial culture reported the growth of predominant one or two microorganisms at ≥10^4^ CFU/ml as possible cause of the suspected UTI. Growth of <10^4^ CFU/ml or growth without predominant microorganism was considered inconclusive for UTI. NGS of the 16S-23S rRNA region identified from 1 to 47 different bacterial species in each PCR-positive sample (Table 1). There was an observed association between the increasing number of bacterial genera/species identified using NGS of 16S-23S rRNA region and decreasing CFU value, median: ≥10^5^ CFU – 2 species/genera; 10^4^ CFU – 5 species/genera; 10^3^ CFU – 5 species/genera; 10^2^ CFU – 20 species/genera. Twenty-nine samples were reported as clinically significant. Among them NGS of 16S-23S rRNA region identified only one bacterial species in 11 samples (UR34, UR35, UR37-UR44, UR50). In the majority of these cases (n = 10) pathogenic species identified by NGS of 16S-23S rRNA region were also identified as the cause of infection by conventional culture methods. The only exception was sample UR44, for which NGS of the 16S-23S rRNA identified solely *K*. *pneumoniae*, but culture also identified *Proteus mirabilis* with 10^3^ CFU/ml. In 12 samples, in which 2 or more species were identified by NGS of the 16S-23S rRNA region (UR3-UR5, UR7-UR9, UR33, UR46, UR48, UR49, UR53, UR55), contigs representing the culture identified pathogenic species consisted of the highest number of reads (53.7–99.1% of all reads). For 3 samples (UR2, UR9, UR59) NGS of the 16S-23S rRNA region showed improved identification compared to conventional culture. In sample UR2 conventional identification showed only the presence of *P*. *mirabilis*, however the NGS-based method also revealed the presence of the genetically related species; *Proteus vulgaris*. In the sample UR9, *Klebsiella oxytoca* was identified by NGS of 16S-23S rRNA region additionally to *E*. *coli*. Colonies produced by these two microorganisms can be difficult to distinguish due to similar morphology on agar plates. In the sample UR59 possible misidentification by conventional culture method could occur. In this sample *K*. *pneumoniae* was identified by Vitek MS and *Klebsiella variicola* by NGS of 16S-23S rRNA region. It was shown very recently, since *K*. *variicola* is closely related to *K*. *pneumoniae*, it is difficult to distinguish between these two species by commonly used methods, including Vitek MS^9^. In the remaining 4 positive samples (UR1, UR36, UR52 and UR54) bacterial species that were identified by conventional culture methods as the cause of infection were also found by the NGS of 16S-23S rRNA method but were not predominant. Usually, only contigs containing commensal organisms consisted of higher number of reads. The urine samples classified by culture as “no clinical significance” (n = 31) contained numerous bacterial species, which probably represented the commensal flora. PCR-negative samples (n = 13) were only those samples with a growth density of 10^2^ CFU/ml.

**Table 1:** Bacterial identification results from 60 urine samples based on culture and NGS of 16S-23S rRNA region.

Sample	Reported cause of UTI by conventional culture identification	Growth CFU/ml	Identification of additional colonies by culture methods	Species content by NGS of 16S-23S rRNA region (% of total reads)
UR1	*Pseudomonas aeruginosa*	10^5^		*Lactobacillus gasseri* (97.2%), *Pseudomonas aeruginosa* (2.4%), *Corynebacterium amycolatum* (0.4%)
UR2	*Proteus mirabilis*	10^4^		*Proteus vulgaris* (70.3%), *Proteus mirabilis* (29.7%)
UR3	*Escherichia coli*	10^5^		*Escherichia coli* (99.1%), *Lactobacillus delbrueckii* (0.9%)
UR4	*Escherichia coli*	10^5^		*Escherichia coli* (99.1%), *Peptoniphilus lacrimalis* (0.3%), *Bacteroides sp*. (0.6%)
UR5	*Escherichia coli*	10^5^		*Escherichia coli* (93.2%), *Undibacterium oligocarboniphilum* (6.3%), *Pseudomonas saccharophila* (0.3%), *Phenylobacterium sp* (0.2%)
UR6	No clinical significance	10^4^	*Bifidobacterium sp*	*Actinobaculum schaalii* (100%)
UR7	*Escherichia coli*	10^5^		*Escherichia coli* (80.7%), *Lactobacillus crispatus* (19.3%)
UR8	*Escherichia coli*	10^5^		*Escherichia coli* (98.7%), *Enterococcus faecalis* (1.0%), *Aerococcus sanguinicola* (0.3%)
UR9	*Escherichia coli*	10^5^		*Escherichia coli* (53.7%), *Klebsiella oxytoca* (43.6%), *Staphylococcus aureus* (2.4%), *Enterococcus faecalis* (0.3%)
	*Staphylococcus aureus*	10^5^		
UR10	No clinical significance	10^2^		*Ezakiella peruensis* (28,6%), *Fenollaria massiliensis* (4,1%), *Helcococcus sp*. (2,6%), *Peptoniphilus duerdenii* (2,6%), *Mobiluncus curtisii* (2,4%), *Varibaculum cambriense* (2,2%), *Peptoniphilus harei* (1,3%), *Actinobaculum urinale* (0,8%), *Peptoniphilus lacrimalis* (0,8%), *Propionimicrobium sp*. (0,7%), *Facklamia sp*. (0,7%), *Finegoldia magna* (0,6%), *Anaerococcus obesiensis* (0,6%), *Anaerococcus prevotii* (0,4%), *Anaerococcus degenerii* (0,4%), *Actinobaculum sp*. (0,4%), *Aerococcus urinae* (0,3%), *Fastidiosipila sanguinis* (0,2%), *Fastidiosipila sanguinis* (0,2%), *Bacteroides coagulans* (0,2%), Unidentified species (50,2%)
UR11	No clinical significance	10^2^	*Staphylococcus epidermidis*	No PCR product
UR12	No clinical significance	10^3^	*Proteus mirabilis*	*Proteus mirabilis* (75.5%), *Proteus vulgaris* (17.5%), *Undibacterium oligocarboniphilum* (4.8%), *Aerococcus urinae* (1.2%), *Corynebacterium striatum* (0.3%), *Pseudomonas saccharophila* (0.3%), *Enterococcus faecalis* (0.2%), *Ralstonia pickettii* (0.1%)
UR13	No clinical significance	10^2^		No PCR product
UR14	No clinical significance	10^2^		*Undibacterium oligocarboniphilum* (36.4%), *Fenollaria massiliensis* (15.6%), *Mobiluncus curtisii* (10.6%), *Peptoniphilus lacrimalis* (5.9%), Unidentified species (5.3%), *Peptostreptococcus anaerobius* (4.4%), *Peptoniphilus koenoeneniae* (4.1%), *Pseudomonas saccharophila* (3.1%), *Atopobium deltae* (3.7%), *Candidatus Peptoniphilus massiliensis* (2.6%), *Anaerococcus sp*. (1.8%), *Jonquetella anthropi* (1.1%), *Peptoniphilus harei* (1.1%), *Streptococcus anginosus* (0.8%), *Ralstonia pickettii* (0.7%), *Dialister propionicifaciens* (0.6%), *Methylobacterium oryzae* (0.6%), *Asinibacterium lactis* (0.5%), *Methylobacterium jeotgali* (0.4%), *Peptoniphilus duerdenii* (0.4%), *Fusobacterium nucleatum* (0.3%)
UR15	No clinical significance	10^2^	*Streptococcus anginosus*	*Unidentified species* (28.3%), *Undibacterium oligocarboniphilum* (13.1%), *Peptoniphilus lacrimalis* (11.5%), *Mobiluncus curtisii* (9.5%), *Streptococcus anginosus* (8.3%), *Fusobacterium nucleatum* (3.8%), *Propionimicrobium lymphophilum* (2.1%), *Varibaculum cambriense* (2.0%), *Dialister sp*. (1.9%), *Atopobium deltae* (1.2%), *Facklamia hominis* (1.1%), *Pseudomonas saccharophila* (1.1%), *Dialister propionicifaciens* (0.9%), *Faecalibacterium prausnitzii* (0.9%), *Parvimonas micra* (0.9%), *Fenollaria massiliensis* (0.9%), *Prevotella disiens* (0.9%), *Peptoniphilus harei* (0.8%), *Actinobaculum massiliense* (0.7%), *Dialister succinatiphilus* (0.7%), *Gemmiger formicilis* (0.7%), *Corynebacterium pyruviciproducens* (0.6%), *Mycoplasma spermatophilum* (0.6%), *Actinobaculum sp*. (0.5%), *Asinibacterium lactis* (0.5%), *Atopobium vaginae* (0.5%), *Mobiluncus curtisii* (0.5%), *Alistipes onderdonkii* (0.4%), *Anaerococcus sp*. (0.4%), *Bacteroides massiliensis* (0.4%), *Anaerococcus lactolyticus* (0.3%), *Anaerococcus obesiensis* (0.3%), *Anaerococcus prevotii* (0.3%), *Finegoldia magna* (0.3%), *Methylobacterium jeotgali* (0.3%), *Peptoniphilus duerdenii* (0.3%), *Peptoniphilus massiliensis* (0.3%), *Ralstonia pickettii* (0.3%), *Treponema refringens* (0.3%), *Actinomyces turicensis* (0.2%), *Bacteroides sp*. (0.2%), *Dialister micraerophilus* (0.2%), *Eubacterium hallii* (0.2%), *Peptoniphilus koenoeneniae* (0.2%), *Peptoniphilus obesi* (0.2%), *Phascolarctobacterium succinatutens* (0.2%), *Porphyromonas bennonis* (0.2%)
UR16	No clinical significance	10^2^		No PCR product
UR17	No clinical significance	10^2^		*Atopobium deltae* (32.6%), *Undibacterium oligocarboniphilum* (19.4%), *Lactobacillus iners* (16.4%), *Unidentified species* (10.5%), *Peptoniphilus coxii* (7.5%), *Anaerococcus lactolyticus* (2.7%), *Parvimonas micra* (2.2%), *Peptoniphilus lacrimalis* (1.8%), *Pseudomonas saccharophila* (1.6%), *Peptoniphilus grossensis* (1.0%), *Aerococcus christensenii* (0.9%), *Peptoniphilus duerdenii* (0.9%), *Peptoniphilus harei* (0.5%), *Actinobaculum schaalii* (0.4%), *Ralstonia pickettii* (0.4%), *Solobacterium moorei* (0.4%), *Asinibacterium lactis* (0.3%), *Propionibacterium acnes* (0.2%), *Varibaculum cambriense* (0.2%)
UR18	No clinical significance	10^2^		No PCR product
UR19	No clinical significance	10^2^		*Undibacterium oligocarboniphilum* (28.1%), *Sneathia sanguinegens* (18.3%), *Lactobacillus iners* (16.5%), *Atopobium vaginae* (4.3%), *Aerococcus christensenii* (4.0%), *Ureaplasma urealyticum* (3.5%), *Pelomonas saccharophila* (1.8%), *Ralstonia pickettii* (1.7%), *Megasphaera indica* (1.4%), *Streptococcus anginosus* (0.9%), *Methylobacterium radiotolerans* (0.6%), *Prevotella amnii* (0.6%), *Methylobacterium aerolatum* (0.5%), *Dialister micraerophilus* (0.4%), *Asinibacterium lactis* (0.2%), *Brevundimonas intermedia* (0.2%), Unidentified species (16.8%)
UR20	No clinical significance	10^2^		No PCR product
UR21	No clinical significance	10^2^		No PCR product
UR22	No clinical significance	10^2^		*Lactobacillus crispatus* (78.5%), *Undibacterium oligocarboniphilum* (14.4%), *Pseudomonas saccharophila* (2.3%), *Undibacterium ligocarboniphilum* (1.5%), *Ralstonia pickettii* (1.0%), *Asinibacterium lactis* (0.4%), *Lactobacillus vaginalis* (0.3%), *Staphylococcus capitis* (0.2%), *Methylobacterium oryzae* (0.1%), Unidentified species (1.3%)
UR23	No clinical significance	10^2^		No PCR product
UR24	No clinical significance	10^3^	*Staphylococcus epidermidis*, *Streptococcus anginosus*	*Lactobacillus jensenii* (98.5%), *Finegoldia magna* (0.5%), *Streptococcus anginosus* (0.5%), *Anaerococcus obesiensis* (0.3%), *Propionimicrobium lymphophilum* (0.2%)
UR25	No clinical significance	10^4^	*Staphylococcus haemolyticus*, *Proteus mirabilis*, *Escherichia coli*	*Lactobacillus iners* (77.8%), *Acinetobacter lwoffii* (12.3%), *Acinetobacter radioresistens* (3.8%), *Escherichia coli* (3.0%), *Proteus mirabilis* (1.1%), *Enterococcus faecalis* (0.5%), *Peptoniphilus harei* (0.4%), *Streptococcus agalactiae* (0.4%), *Finegoldia magna* (0.2%), *Proteus vulgaris* (0.2%), *Corynebacterium sp*. (0.1%), *Pseudomonas stutzeri* (0.1%), *Streptococcus sp*. (0.1%)
UR26	No clinical significance	10^2^	*Staphylococcus hominis*	No PCR product
UR27	No clinical significance	10^2^	*Staphylococcus hominis*	*Lactobacillus iners* (84.1%), *Lactobacillus jensenii* (11.1%), *Lactobacillus sp*. (2.8%), *Undibacterium oligocarboniphilum* (0.9%), *Staphylococcus hominis* (0.5%), *Lactobacillus vaginalis* (0.2%), *Pseudomonas saccharophila* (0.2%), *Asinibacterium lactis* (0.1%), *Corynebacterium tuberculostearicum* (0.1%)
UR28	No clinical significance	10^3^	*Enterococcus faecalis*, *Streptococcus mitis/oralis*	No PCR product
UR29	No clinical significance	10^2^		No PCR product
UR30	No clinical significance	10^2^		No PCR product
UR31	No clinical significance	10^2^		No PCR product
UR32	No clinical significance	10^2^		*Lactobacillus iners* (98.6%), *Lactobacillus jensenii* (1.4%)
UR33	*Pseudomonas aeruginosa*	10^5^		*Pseudomonas aeruginosa* (99.7%), *Peptostreptococcaceae sp*. (0.2%), *Anaerococcus degenerii* (0.1%)
UR34	*Escherichia coli*	10^4^		*Escherichia coli* (100%)
UR35	*Escherichia coli*	10^5^		*Escherichia coli* (100%)
UR36	*Proteus mirabilis*	10^5^		*Unidentified species* (61.4%), *Peptoniphilus harei* (18.0%), *Proteus mirabilis* (16.3%), *Aerococcus urinae* (4.2%), *Staphylococcus aureus* (0.1%)
UR37	*Escherichia coli*	10^5^		*Escherichia coli* (100%)
UR38	*Escherichia coli*	10^5^		*Escherichia coli* (100%)
UR39	*Klebsiella pneumoniae*	10^5^		*Klebsiella pneumoniae* (100%)
UR40	*Pseudomonas aeruginosa*	10^4^		*Pseudomonas aeruginosa* (100%)
UR41	*Klebsiella pneumoniae*	10^5^		*Klebsiella pneumoniae* (100%)
UR42	*Escherichia coli*	10^4^		*Escherichia coli* (100%)
UR43	*Klebsiella pneumoniae*	10^5^		*Klebsiella pneumoniae* (100%)
UR44	*Klebsiella pneumoniae*	10^5^		*Klebsiella pneumoniae* (100%)
	*Proteus mirabilis*	10^3^		
UR45	No clinical significance	10^3^	*Streptococcus mitis/oralis*, *Escherichia coli*, *Staphylococcus aureus*, *Staphylococcus epidermidis*	*Ureaplasma parvum* (42.2%), *Prevotella sp*. (27.0%), *Prevotella bivia* (10.8%), *Anaerococcus lactolyticus* (7.7%), *Peptostreptococcus anaerobius* (6.4%), *Peptoniphilus sp*. (1.7%), *Finegoldia magna* (1.5%), *Streptococcus oralis* (1.5%), *Escherichia coli* (0.6%), *Lactobacillus crispatus* (0.4%), *Staphylococcus aureus* (0.3%)
UR46	*Enterococcus feacalis*	10^4^	*Staphylococcus epidermidis*, *Streptococcus anginosus*, *Staphylococcus capitis*	*Enterococcus faecalis* (99.7%), *Lactobacillus iners* (0.3%)
UR47	No clinical significance.	10^4^	*Gordonia alkalivorans*, *Sphingomonas trueperi*, *Staphylococcus schleiferi*, *Corynebacterium sputorum*	*Aerococcus sanguinicola* (92,5%), *Aerococcus sp*. (4,4%), *Aerococcus urinae* (1,8%), *Peptoniphilus harei* (0,9%), *Lactobacillus iners* (0,3%)
UR48	*Staphylococcus aureus*	10^5^	*Klebsiella pneumoniae*	*Staphylococcus aureus* (99,8%), *Escherichia coli* (0,2%)
UR49	*Staphylococcus epidermidis*	10^5^		*Staphylococcus epidermidis* (97.6%), *Staphylococcus sp*. (1.3%), *Gardnerella vaginalis* (1.1%)
UR50	*Enterococcus feacalis*	10^5^		*Enterococcus faecalis* (100%)
UR51	No clinical significance	10^3^	*Staphylococcus epidermidis*	*Staphylococcus epidermidis* (100%)
UR52	*Streptococcus aggalactiae*	10^4^	*Staphylococcus warneri*	*Actinobaculum schaalii* (54.4%), *Streptococcus agalactiae* (45.6%)
UR53	*Escherichia coli*	10^5^		*Escherichia coli* (*70*.*1%*), *Aerococcus urinae* (*22*.*5%*), *Enterococcus sp*. (*7*.*5%*)
	*Enterococcus feacalis*	10^5^		
UR54	*Escherichia coli* ^A^	10^4^		*Lactobacillus gasseri* (53.2%), *Escherichia coli* (41.8%), *Streptococcus pasteurianus* (4.7%), *Enterococcus sp*. (0.3%)
	*Escherichia coli* ^A^	10^4^		
UR55	*Klebsiella pneumoniae*	10^5^		*Klebsiella pneumoniae* (74.0%), *Actinotignum sanguinis* (21.6%), *Lactobacillus crispatus* (2.6%), *Aerococcus urinae* (1.8%)
UR56	No clinical significance	10^2^		Eukaryotic DNA (85.0%), *Anaerococcus sp*. (6.5%), *Staphylococcus epidermidis* (3.3%), *Klebsiella pneumoniae* (2.6%), *Aerococcus urinae* (1.7%), *Escherichia coli* (0.8%)
UR57	No clinical significance	10^2^		No PCR product
UR58	No clinical significance	10^2^	*Staphylococcus epidermidis*	Peptoniphilus sp. (22.1%), Anaerococcus sp. (19.8%), *Fusobacterium nucleatum* (18.5%), *Unidentified* (17.3%), *Streptococcus anginosus* (10.8%), *Dialister sp*. (7.3%), *Aerococcus urinae* (2.0%), *Sneathia sp*. (0.6%), *Lactobacillus iners* (0.4%), *Lactobacillus sp*. (0.4%), *Dialister micraerophilus* (0.4%), *Fenollaria sp*. (0.4%)
UR59	*Klebsiella pneumoniae*	10^5^		*Klebsiella variicola* (89.4%), *Klebsiella sp* (10.6%)
UR60	No clinical significance	10^2^		*Staphylococcus epidermidis* (27.0%), *Ferruginibacter sp*. (14.7%), *Corynebacterium aurimucosum* (11.1%), *Methylobacterium jeotgaliv* (10.6%), *Acidobacteria* (8.8%), *Dokdonella fugitiva* (5.2%), *Methylobacterium oryzae* (5.1%), *Paracoccus marinus* (4.0%), *Marmoricola aurantiacus* (3.4%), *Flavobacteriaceae* (2.6%), *Bacteroides ureolyticus* (1.6%), *Nocardioides sp*. (1.0%), *Ferruginibacter sp*. (0.9%), *Pelomonas saccharophila* (0.8%), *Ferruginibacter sp*. (0.8%), *Propionibacterium acnes* (0.7%), *Campylobacter ureolyticus* (0.6%), *Gemmatimonadetes* (0.4%), *Niabella sp*. (0.4%), *Corynebacterium tuberculostearicum* (0.3%),

^A^Stains revealed different resistance patterns.

### Blood stream infections

Among the 23 positive blood culture samples, conventional culture methods and the NGS of the 16S-23S rRNA region approach produced the same identifications for 20 samples (Table 2). For 3 samples (BC11, 19 and 22) identification of bacterial organisms to the species level was improved by the NGS-based method. In case of sample BC11 conventional culture methods were only able to identify the microorganism to the genus level of *Bacteroides*. In the other two samples, the contigs representing *Streptococcus lutetiensis* (BC19) and *Bacteroides dorei* (BC22) showed 99.9% (4283/4284) and 100% (4433/4433) sequence homology, respectively, during BLAST analysis of the 16S-23S rRNA region. Only a 16S rRNA sequence for *Streptococcus infantis* was available in the GenBank database showing 95.4% (1413/1481) similarity and for *Bacteroides vulgatus* the whole 16S-23S rRNA region shared only 96.5% (4286/4443) similarity to the obtained contig sequence.

**Table 2:** Bacterial identification results from 23 positive blood culture bottles based on culture and NGS of 16S-23S rRNA region.

Sample	Patient	Bottle	Culture (Maldi-TOF MS)	NGS of 16S-23S rRNA region (% of total reads)
BC01	Patient A	anaerobic	*Escherichia coli*	*Escherichia coli* (100%)
BC02	Patient B	aerobic	*Streptococcus dysgalactiae*	*Streptococcus dysgalactiae* (100%)
BC03	Patient C	anaerobic	*Klebsiella oxytoca*	*Klebsiella oxytoca* (100%)
BC05	Patient D	aerobic	*Staphylococcus heamolyticus*	*Staphylococcus haemolyticus* (100%)
BC06	Patient E	anaerobic	*Staphylococcus hominis*	*Staphylococcus hominis* (100%)
BC07	Patient F	aerobic	*Staphylococcus capitis*	*Staphylococcus capitis* (100%)
BC08	Patient G	anaerobic	*Streptococcus pneumoniae*	*Streptococcus pneumoniae* (100%)
BC09	Patient H	aerobic	*Staphylococcus epidermidis*	*Staphylococcus epidermidis* (100%)
BC10	Patient H	anaerobic	*Staphylococcus hominis*	*Staphylococcus hominis* (100%)
BC11	Patient I	anaerobic	*Bacteroides sp*.	*Bacteroides fragilis* (100%)
BC12	Patient J	aerobic	*Staphylococcus hominis*	*Staphylococcus hominis* (100%)
BC13	Patient K	aerobic	*Staphylococcus aureus*	*Staphylococcus aureus* (100%)
BC14	Patient L	aerobic	*Klebsiella oxytoca*	*Klebsiella oxytoca* (100%)
BC15	Patient M	anaerobic	*Streptococcus pneumoniae*	*Streptococcus pneumoniae* (100%)
BC16	Patient N	aerobic	*Escherichia coli*	*Escherichia coli* (100%)
BC17	Patient O	anaerobic	*Staphylococcus aureus*	*Staphylococcus aureus* (100%)
BC18	Patient P	anaerobic	*Streptococcus pneumoniae*	*Streptococcus pneumoniae* (100%)
BC19	Patient Q	aerobic	*Escherichia coli*, *Streptococcus infantis*	*Escherichia coli* (69.3%), *Streptococcus lutetiensis* (30.7%)
BC20	Patient Q	anaerobic	*Escherichia coli*	*Escherichia coli* (100%)
BC21	Patient R	aerobic	*Escherichia coli*	*Escherichia coli* (100%)
BC22	Patient R	anaerobic	*Bacteroides vulgatus*	*Bacteroides dorei* (100%)
BC23	Patient S	aerobic	*Staphylococcus hominis*	*Staphylococcus hominis* (100%)
BC24	Patient S	aerobic	*Staphylococcus epidermidis*	*Staphylococcus epidermidis* (100%)

### Orthopedic infections

Of the 21 clinical samples of orthopedic patients 18 were found to be culture negative and three samples were not cultured. In 5 samples (KM2, KM7, KM12, KM16 and KM20) the NGS-based method was able to detect only eukaryotic DNA and in 2 samples (KM8 and KM10) it yielded non-interpretable results most likely because of template degradation. In the remaining 14 samples the number of microorganisms detected in the orthopedic samples ranged from 1 to 3 different genera/species (Table 3). The only exception was sample KM9 in which 11 different genera/species were found. In our study, orthopedic samples had a low amount of starting material, so they were especially prone to be swamped by the contaminating DNA and result in misleading results. All negative controls contained *Pseudomonas fluorescens*. In a single negative control, *Propionibacterium acnes* was also found. Therefore, in orthopedic samples *P*. *fluorescens* (present in KM11 and KM19) and *P*. *acnes* (KM1, KM5, KM9, KM11, KM13, KM14, KM18 and KM19) were regarded as contamination introduced during sample preparation. Moreover, several bacterial genera previously reported as contamination of DNA extraction kits, PCR and other laboratory reagents were absent in all negative controls but present at orthopedic samples, including *Acinetobacter*, *Bacillus*, *Chryseobacterium*, *Enhydrobacter*, *Paracoccus*, *Psychrobacter* and *Undibacterium* ^10^. These bacteria can also be potentially considered as contaminations of the 16–23S rRNA NGS process introduced during sample processing.

**Table 3:** Bacterial identification results from 21 clinical orthopedic samples based on culture and NGS of 16S-23S rRNA region.

Sample	Patient	Material	Culture	NGS of 16–23S rRNA region (% of total reads)
KM1	Patient A	biopsy (tissue)	Negative	*Propionibacterium acnes* (9.1%)^A^, *Haemophilus parainfluenzae* (2.3%), eukaryotic DNA (88.6%)
KM2	Patient A	punctate (fluid)	Negative	eukaryotic DNA (100%)
KM3	Patient A	punctate (fluid)	Negative	*Sediminibacterium salmoneum* (0.3%), eukaryotic DNA (99.7%)
KM4	Patient A	punctate (fluid)	Negative	*Gemella sanguinis* (1.3%), *Haemophilus parainfluenzae* (1.0%), eukaryotic DNA (97.7%)
KM5	Patient A	punctate (fluid)	Negative	*Herminiimonas sp*. (10.5%), *Propionibacterium acnes* (9.7%)^A^, *Moraxella catarrhalis* (7.5%), eukaryotic DNA (72.3%)
KM6	Patient B	pus	Negative	*Streptococcus intermedius* (100%)
KM7	Patient C	biopsy (tissue)	Negative	eukaryotic DNA (100%)
KM8	Patient C	biopsy (tissue)	Negative	No identification
KM9	Patient D	joint puncture (fluid)	Negative	*Enhydrobacter aerosaccus* (49.8%)^B^, *Acinetobacter septicus* (18.1%)^B^, *Moraxella osloensis* (14.0%), *Staphylococcus sp*. (5.8%), *Rheinheimera soli* (3.1%), *Staphylococcus epidermidis* (2.6%), *Psychrobacter sp*. (2.4%)^B^, *Propionibacterium acnes* (1.3%)^A^, *Alkanindiges sp*. (0.6%), *Acinetobacter sp*. (0.4%)^B^, *Chryseobacterium sp*. (0.3%)^B^
KM10	Patient D	joint puncture (fluid)	Negative	No identification.
KM11	Patient D	biopsy (tissue)	Negative	*Propionibacterium acnes* (9.8%)^A^, *Bacillus nealsonii* (6.7%)^B^, *Pseudomonas fluorescens* (0.6%)^A^, eukaryotic DNA (82.9%)
KM12	Patient D	biopsy (tissue)	Negative	eukaryotic DNA (100%)
KM13	Patient D	biopsy (tissue)	Negative	*Undibacterium oligocarboniphilum* (3.5%)^B^, *Propionibacterium acnes* (0.7%)^A^, eukaryotic DNA (95.9%)
KM14	Patient D	biopsy (tissue)	Negative	*Propionibacterium acnes* (1.4%)^A^, eukaryotic DNA (98.6%)
KM15	Patient D	biopsy (tissue)	Negative	*Veillonella parvula* (0.9%), eukaryotic DNA (99.1%)
KM16	Patient D	biopsy (tissue)	Negative	eukaryotic DNA (100%)
KM17	Patient E	blood	n.d.	*Bacillus cereus* (0.5%)^B^, eukaryotic DNA (99.5%)
KM18	Obduction material A	formaline captured, biopt (tissue)	n.d.	*Propionibacterium acnes* (64.4%)^A^, *Staphylococcus epidermidis* (25.4%), *Paracoccus sanguinis* (10.1%)^B^
KM19	Obduction material B	formaline captured, lung biopt (tissue)	n.d.	*Staphylococcus epidermidis* (36.0%), *Propionibacterium acnes* (34.6%)^A^, *Pseudomonas fluorescens* (29.4%)^A^
KM20	Patient F	joint puncture (fluid)	Negative	eukaryotic DNA (100%)
KM21	Patient F	biopsy (tissue)	Negative	*Acinetobacter sp*. (18.6%)^B^, *Paucibacter sp*. (12.8%), *Herminiimonas arsenicoxydans* (5.2%), eukaryotic DNA (63.4%)

^A^Species present in negative control(s) and regarded as contamination introduced during sample preparation. ^B^Genus absent in negative controls but previously reported as contamination of DNA extraction kits, PCR and other laboratory reagents^10^.

### Reproducibility

To assess the reproducibility of the method based on NGS of the 16S-23S rRNA region, 6 urine samples were used: 2 samples with bacterial count level at 10^2^ CFU/ml, single samples with bacterial count levels at 10^4^ and 10^3^ CFU/ml, and 2 samples with bacterial count level at ≥ 10^5^ CFU/ml (Table 4). The samples were analyzed in triplicate by 3 different operators, including independent PCR amplification, PCR product purification, and NGS library preparation. Subsequently, two independent MiSeq sequencing runs were conducted. The first replicate of each sample was sequenced in the first run, while the second and third replicate of the tested samples were sequenced in parallel in the second run. When an organism identified was represented by 5% or more reads in at least one replicate of a sample, this organism was always detected in its remaining two replicates (Table 4). The only exception was *Undibacterium oligocarboniphilum*, previously reported as a contaminant in DNA extraction kits, and in PCR and other laboratory reagents^10^. In the samples with bacterial count level at ≥10^5^ CFU/ml, the method achieved 100% of reproducibility with respect to the bacterial composition. Moreover, in all replicates of a sample with a bacterial count level at ≥10^3^ CFU/ml always the same organism was predominant and represented by 50.9–100% of reads.

**Table 4:** Reproducibility of NGS of the 16S-23S region.

Sample (Growth CFU/ml)	Identification	Detection Frequency (n)	Average (% reads)	Range (% reads)^B^	SD^B^
UR12 (10^3^)	*Proteus mirabilis*	3	90.0	75.5–97.9	12.6
	*Proteus vulgaris*	3	6.8	0.6–17.5	9.3
	*Aerococcus urinae*	3	1.3	1.1–1.5	0.2
	*Undibacterium oligocarboniphilum* ^A^	1	4.8	n.a.	n.a.
	*Corynebacterium striatum*	1	0.3	n.a.	n.a.
	*Pseudomonas saccharophila*	1	0.3	n.a.	n.a.
	*Enterococcus faecalis*	1	0.2	n.a.	n.a.
	*Ralstonia pickettii* ^A^	1	0.1	n.a.	n.a.
UR14 (10^2^)	Unidentified species	3	23.6	5.3–42.0	18.4
	*Peptoniphilus lacrimalis*	3	17.9	5.9–33.7	14.3
	*Fenollaria massiliensis*	3	13.5	11.6–15.6	2.0
	*Peptostreptococcus anaerobius*	3	9.5	4.4–13.1	4.5
	*Mobiluncus curtisii*	3	6.0	2.1–10.6	4.3
	*Anaerococcus sp*.	3	3.6	1.8–6.6	2.6
	*Atopobium deltae*	3	2.8	2.3–3.7	0.8
	*Dialister propionicifaciens*	3	0.8	0.6–1.2	0.3
	*Jonquetella anthropi*	3	0.6	0.3–1.1	0.4
	*Tissierella sp*.	2	2.3	2.0–2.6	0.4
	*Moryella sp*.	2	0.8	0.1–1.4	0.9
	*Fusobacterium nucleatum*	2	0.5	0.3–0.7	0.3
	*Ezakiella sp*.	2	0.2	0.1–0.2	0.1
	*Undibacterium oligocarboniphilum* ^A^	1	36.4	n.a.	n.a.
	*Peptoniphilus koenoeneniae*	1	4.1	n.a.	n.a.
	*Prevotella bivia*	1	3.4	n.a.	n.a.
	*Pseudomonas saccharophila*	1	3.1	n.a.	n.a.
	*Candidatus Peptoniphilus massiliensis*	1	2.6	n.a.	n.a.
	*Peptoniphilus harei*	1	1.1	n.a.	n.a.
	*Streptococcus anginosus*	1	0.8	n.a.	n.a.
	*Ralstonia pickettii* ^A^	1	0.7	n.a.	n.a.
	*Methylobacterium oryzae* ^A^	1	0.6	n.a.	n.a.
	*Streptococcus sp*.	1	0.6	n.a.	n.a.
	*Asinibacterium lactis*	1	0.5	n.a.	n.a.
	*Olsenella sp*.	1	0.5	n.a.	n.a.
	*Methylobacterium jeotgali* ^A^	1	0.4	n.a.	n.a.
	*Peptoniphilus duerdenii*	1	0.4	n.a.	n.a.
	*Filifactor sp*.	1	0.2	n.a.	n.a.
	*Proteus mirabilis*	1	0.2	n.a.	n.a.
	*Actinotignum sp*.	1	0.2	n.a.	n.a.
	*Bacteroides sp*.	1	0.2	n.a.	n.a.
	*Shigella flexneri*	1	0.1	n.a.	n.a.
	*Casaltella sp*.	1	0.1	n.a.	n.a.
	*Howardella sp*.	1	0.1	n.a.	n.a.
	*Propionibacterium acnes*	1	0.1	n.a.	n.a.
UR22 (10^2^)	*Lactobacillus crispatus*	3	92.8	78.5–100	12.4
	*Undibacterium oligocarboniphilum* ^A^	1	14.4	n.a.	n.a.
	*Pseudomonas saccharophila*	1	2.3	n.a.	n.a.
	*Undibacterium ligocarboniphilum* ^A^	1	1.5	n.a.	n.a.
	Unidentified species	1	1.3	n.a.	n.a.
	*Ralstonia pickettii* ^A^	1	1.0	n.a.	n.a.
	*Asinibacterium lactis*	1	0.4	n.a.	n.a.
	*Lactobacillus vaginalis*	1	0.3	n.a.	n.a.
	*Staphylococcus capitis*	1	0.2	n.a.	n.a.
	*Methylobacterium oryzae* ^A^	1	0.1	n.a.	n.a.
UR67 (10^5^)	*Escherichia coli*	3	66.1	62.4–70.1	3.9
	*Aerococcus urinae*	3	26.8	22.5–29.5	3.8
	*Enterococcus sp*.	3	8.5	7.5–9.2	0.9
UR68 (10^4^)	*Lactobacillus gasseri*	3	55.5	50.9–62.4	6.1
	*Escherichia coli*	3	36.9	34.4–41.8	4.3
	*Streptococcus pasteurianus*	3	5.2	2.3–8.7	3.2
	*Enterococcus sp*.	3	1.0	0.3–2.0	0.9
	*Veillonella sp*.	2	0.2	0.2–0.2	0.0
	Unidentified species	1	3.6	n.a.	n.a.
	*Facklamia sp* ^A^	1	0.2	n.a.	n.a.
UR69 (10^5^)	*Klebsiella pneumoniae*	3	77.5	73.1–85.5	6.9
	*Aerococcus urinae*	3	12.8	1.8–25.1	11.7
	*Actinotignum sanguinis*	3	8.6	1.3–21.6	11.3
	*Lactobacillus crispatus*	3	1.0	0.1–2.6	1.4

^A^Bacterial microorganism previously reported as contamination of DNA extraction kits, PCR and other laboratory reagents^10^. ^B^n.a., not assigned.

## Discussion

As defined in the CLSI guidelines, a species identification can be assigned when the max score is 99% or higher and if the sequence similarity between best and second best species are greater than 0.5% using DNA target sequencing^11^. However, species identification using CLSI’s criteria for the 16S rRNA gene sequences was often weak because the criteria of distance scores greater than 0.5% to the next closest species was not met for most strains. In such instances, only identification to the genus level was feasible. Therefore, Park *et al*.^12^ proposed a modified CLSI (mCLSI) method which was more practical and pragmatic for identification of species based on 16S rRNA sequences than the CLSI method. The mCLSI method assigns bacterial species when the similarity score is 99% or higher but irrespective of the similarity score differences. In our study, we applied the similarity score differences with the next closest species as ≥0.2%, which reflected at least 3 and 9 nucleotides difference by sequencing the 16S rRNA gene or the 16S-23S rRNA region, respectively. It allowed elimination of misidentification of very closely related species like *S*. *oralis* and *S*. *mitis* (Table S1) which in their 16S rRNA gene sequence differ only by 1 nucleotide. Using Sanger sequencing of the 16S rRNA gene, species was assigned with the similarity score 99.4% or higher (Table S1). Using NGS of the 16S-23S rRNA region, in a great majority of cases (n = 61), species were assigned with a similarity score above 99.2%. The exceptional 6 species with lower scores showed a similarity ranging from 96.2% to 98.8%. This could be caused by the facts that (i) a limited number of 16S-23S rRNA sequences were available in databases and (ii) the CLSI guidelines were developed for comparison of gene sequences and did not include the intergenic regions which for some genera can be endowed with higher variation. We believe that with increasing number of deposited 16S-23S rRNA sequences, it will be always possible to assign bacterial species with similarity scores of 99% or higher.

We assigned bacteria to the genus level by NGS of 16S-23S rRNA region in clinical samples when the similarity score was at least 90%. This value was determined based on the results produced during comparison of the four identification methods on pure cultures (Table S1). The 16S-23S rRNA sequence alignments produced the lowest identity for second closest match within the same genus for: Actinobaculum 88% (Table S1; sample 6), Lactobacillus 89% (sample 30), Corynebacterium 91% (samples 14, 17 and 18). We applied the uniform cut-off equal to 90% for all bacteria in clinical samples. However, defining 16S-23S rRNA sequences for all or at least majority species within a genus will create interpretive criteria for defining the genus and probably will vary according to the queried microorganism.

The 16S-23S rRNA sequences can differ by length even among highly genetically related species. It is caused by size variation in 16S-23S rRNA intergenic spacer region (ISR). *Staphylococcus aureus* clonal complex 75 has been recently renamed as *Staphylococcus argenteus* and now is a novel species of the *Staphylococcus* genus^13^. *S*. *argenteus* showed identical or nearly identical 16S and 23S rRNA gene sequences to *S*. *aureus* but differed substantially by the ISR length. The scoring system of BLAST had been designed not to allow large gaps. The BLAST algorithm produced a set of smaller separate alignments, with the longest alignment encompassing the 23S rRNA gene (about 2, 5 kb in size). The lack of an alignment for the full-length sequences allowed distinguishing *S*. *aureus* from *S*. *argenteus*.

In our study all negative controls contained *P*. *fluorescens*. We, however, only found this microorganism in the orthopedic samples (Table 3) and not in the urine and blood samples (Tables 1 and 2). Orthopedic samples were characterized with lower starting microbial material compared to that of the urine and blood samples. This showed that the impact of contaminating sequences is greater in low biomass samples. Moreover, *P*. *fluorescens* had not been associated previously with human infections and its presence can be clearly regarded as contamination. However, in a single negative control, which was introduced to monitor the impact of contaminations on identification procedure of the orthopedic samples, *P*. *acnes* was also identified. *P*. *acnes* is a common human skin-associated organism and had been previously shown as the cause of orthopedic infections^14, 15^. It will be highly important to limit the impact of such contaminations as *P*. *acnes* during samples handling as this is also a clinically significant microorganism and its contribution to an infection can be misinterpreted.

NGS of the 16S-23S rRNA region will provide enhanced information on the presence of microbial DNA within a clinically relevant time frame which is necessary for timely and accurate treatment of infections and therefore key for proper infection and antibiotic resistance control. Data of this study showed that this method correctly identified bacterial species that were identified as the cause of infection by conventional culture methods. In general, in the culture-positive samples a few additional species/genera per sample were identified by the NGS method. However, contigs representing the culture identified pathogenic bacterial species had the highest number of sequence reads. To assess the clinical relevance of the identified species/genera in samples with a low amount of bacterial DNA, a prospective clinical validation study will be carried out including samples of complex patient groups (e.g. orthopedic patients with a clinical suspicion of a prosthetic joint infection) and samples of control groups of patients without a clinical suspicion of an infection.

Currently, NGS of the 16S-23S rRNA region suffers from two major limitations. First limitation is a lack of reference database with the 16S-23S rRNA sequences and a complementary software allowing easy and reliable species identification. Second major limitation of the method is a lack of reference sequences for many bacterial species. We currently work on the development of a reference data set for assigning clinically relevant bacterial species based on the 16S-23S rRNA sequences. The quality and amount of data accumulated in the databases is particularly important for the performance of bacterial identification using sequencing analysis of the 16S-23S rRNA region. Also, this lack of reference 16S-23S rRNA sequences in the GenBank database might have potentially introduced bias in the results when comparing 16S rRNA with 16S-23S rRNA sequences.

As a proteomic tool for microbial identification, MALDI-TOF MS is superior to NGS-based methods in cost and speed. Currently, the total costs of all reagents and consumables for NGS of 16S-23S rRNA region (not including labor) amount to ~70 € per sample with turnaround time of ≤4 days. However, NGS of the 16S-23S rRNA region was culture independent and more significantly discriminative at the species and genus level than MALDI-TOF MS approaches. Moreover, NGS of 16S-23S rRNA region, unlike all other mass spectrometry methods described above, can be extremely useful for identification of rare or unknown bacteria and bacteria with unusual phenotypic profiles.

In summary, the main objective of the present study was to develop a new diagnostic method based on NGS of 16S-23S rRNA region and assess its identification potential. Its resolution power was found to be superior to the other identification approaches commonly used in routine clinical microbiology laboratories; moreover, the method was easy in use. The method correctly identified urinary tract pathogens and blood stream pathogens previously identified as the cause of UTI and blood stream infection (BSI) with conventional culture. NGS of the 16S-23S rRNA region also showed increased sensitivity in the diagnosis of bacterial microorganisms in samples collected from patients suspected of orthopedic infections. In the analyzed samples several bacterial species had been previously reported as the cause of orthopedic infections, including *Haemophilus parainfluenzae* ^16^, *Moraxella osloensis* ^17^ and *S*. *epidermidis* ^18^. Therefore, we conclude that our approach has the potential to increase diagnostic yield and will decrease time to result for detection of unexpected bacterial pathogens and bacterial species compared to current methods, thereby improving targeted antibiotic treatment. Furthermore, there is a huge potential of this method for detection of bacterial pathogens that can not be cultured at all, due to VNC state or due to antibiotics prior to collection of sample. Finally, with our method it will be possible to streamline processes in the laboratory and to implement it in several disciplines, like clinical, environmental and veterinary microbiology. However, this approach needs further validation and determination of its sensitivity. Furthermore, studies focused on the clinical relevance are necessary for determining the applicability of this NGS-based approach in routine diagnostics.

## Materials and Methods

### Ethics

All procedures were carried out according to guidelines and regulations of Certe concerning the use of patient materials for the validation of clinical methods, which are in compliance with the guidelines of the Federation of Dutch Medical Scientific Societies (FDMSS). The project was approved by the Certe medical staff under project submission 3305-0037. Every patient of the Certe is informed that samples taken may be used for research and publication purposes unless they indicate that they do not agree to it. Informed consent was obtained from all individuals or their guardians prior to study participation. All samples were used after performing and completing a conventional microbiological diagnosis and were coded to protect patients confidentiality.

### Bacterial isolates

Pure cultures of 44 difficult to identify clinically bacterial isolates were included. Additionally, a selection of 23 isolates was used, including 15 ATCC strains (Table S1).

### Clinical samples

Sixty fresh urine samples from patients suspected of urinary tract infections (UTI), 23 BacT/ALERT (bioMérieux) positive blood culture bottles from patients suspected of BSI and 21 clinical samples of orthopedic patients were collected. Culture was performed as part of routine diagnostics by the department of Bacteriology at Certe.

### Vitek MS (bioMérieux)

Vitek MS slides were prepared and interpreted according to the manufacturer’s instructions. Strains which did not yield an identification due to unreliable results or bad spectra were repeated once.

### Bruker MS (Bruker)

Bruker Microflex slides were prepared using on-slide extraction and interpreted according to manufacturer’s instructions. Strains which did not yield an identification due to unreliable results or bad spectra were repeated once.

### Bacterial isolates DNA extraction

Blood agar plates were used for culturing the bacterial strains. DNA isolation from pure cultures was performed using the UltraClean Microbial DNA Isolation Kit (Mobio) according to the manufacturer’s instructions.

### Urine DNA extraction

DNA isolation of urine samples was performed using the UltraClean Microbial DNA Isolation Kit (Mobio). Briefly, 500 µl of urine was centrifuged for 30 seconds at 10,000 g. The supernatant was discarded and the pellet reconstituted in 300 µl Microbead solution. Since then the manufacturer’s instructions were followed for DNA extraction.

### Blood culture bottle DNA extraction

DNA from 1.8 ml from positive blood culture bottles was extracted using the BiOstic Bacteraemia DNA Isolation Kit (Mobio) according to the manufactures instructions.

### DNA extraction of the clinical samples of orthopedic patients

The Purelink Genomic DNA purification kit (Invitrogen) was used for DNA extraction of the orthopedic samples. Initial lysis was performed using lysis buffer (0.25 M NaCl, 0.5% SDS, 5x TE, 2.25 U/ml Proteinase K (Roche)). For tissue samples a small piece was digested in 200 µl lysis buffer. For fluidic samples 200 µl was added to 200 µl lysis buffer. Digestion was performed in a thermoshaker at 56 °C under light shaking for 18 hours. 200 µl Purelink Genomic lysis/binding buffer was added to 200 µl of lysed sample and vortexed to create a homogenous solution. 200 µl 96% ethanol was added and the DNA purification protocol was followed according to the manufacturer’s instructions.

### 16S rRNA gene amplification and Sanger sequencing

The DNA of the bacterial isolates and controls were amplified with 0.3 μM primers [LPW57 5′-AGTTTGATCCTGGCTCAG-3′ (nucleotide position 10–27) and LPW58 5′-AGGCCCGGGAACGTATTCAC-3′ (nucleotide position 1370–1389)]^19^ using GoTaq® MDx Hot Start Polymerase and Colorless GoTaq® Flexi Buffer (Promega). PCR amplification was performed on a PTC-100 thermocycler (Bio-Rad) using the following conditions: an initial incubation at 94 °C for 2 min, 25 cycles of 94 °C for 30s, 58 °C for 30s, and 72 °C for 1 min, followed by a final incubation at 72 °C for 5 min. PCR products were purified using DNA Clean & Concentrator (Zymo Research). DNA sequencing was performed by the GATC company (Cologne, Germany) using the PCR primers. 16S rRNA gene sequences were subjected to BLAST analysis against the NCBI nucleotide database. The sequencing data analysis allowed assigning species when the similarity score was 99% or higher and the similarity score differences with the next closest species were equal to or greater than 0.2%, which reflected 3 or more nucleotides. The genus was assigned when the similarity score was 97% or higher. The BLAST analysis of the 16S rRNA gene sequences was verified against the curated leBiBi-QBPP database (https://umr5558-bibiserv.univ-lyon1.fr/lebibi/lebibi.cgi) not revealing any differences in identification.

### Amplification of the 16S-23S rRNA region

The 16S-23S rRNA region was amplified by PCR using forward primer 27F (5′-AGAGTTTGATCMTGGCTCAG-3′), specific for the 16S rRNA gene and adapted from the study by Sergeant *et al*.^20^, and reverse primer 2490R (5′-GACATCGAGGTGCCAAAC-3′), specific for the 23S rRNA gene and slightly modified by truncation of a single nucleotide compared to the original publication by Hunt *et al*.^21^. The amplification of the 16S-23S DNA region was carried out in a 25 µl reaction consisting of 1x Phire hotstart buffer (Thermofisher), 5 mM dNTP’s (Roche), 0.5 µl Phire hotstart II DNA polymerase (Thermofisher), 600 nM of each primer and 5 µl of DNA template. PCR was performed using a Biorad PTC-200 thermocycler. An initial denaturation of 98 °C at 30 sec was followed by 25 cycles of 98 °C for 30 sec, 66 °C for 30 sec and 72 °C for 2 minutes with a final extension of 72 °C for 1 min. To increase PCR sensitivity in clinical samples the number of cycles was increased to 35. PCR products were purified using the Qiaquick PCR purification kit (Qiagen) according to the manufacturer’s instructions.

### NGS library preparation and Illumina MiSeq sequencing

For library preparation, the Nextera XT DNA Sample Preparation Kit (Illumina) was used according to the manufacturer’s instructions. Briefly, the purified PCR amplicons quantified with a Qubit 2.0 Fluorometer (Thermofisher) were diluted to 0.2 ng/µl and a total of 1 ng of DNA was tagmented at 55 °C for 5 min. PCR amplification to introduce Illumina index sequences was performed in PCR strip tubes in a BioRad T100 thermocycler. Size distribution of fragments was estimated with a 2200 TapeStation using the Agilent D1000 High Sensitivity kit according to the manufacturer’s instructions. Fragments of 200 to 1000 bp were obtained. The library DNA fragments were size selected and purified using AMPure XP beads (Beckman Coulter, Inc.). The indexed libraries were normalized, pooled and loaded onto an Illumina MiSeq reagent cartridge using MiSeq reagent kit v3 and 600 cycles. The paired-end 2 × 300 bp sequencing was run on an Illumina MiSeq sequencer.

### Sequencing of the 16S-23S rRNA region and data analysis

NGS generated 25,000–50,000 or 1–2 million sequencing reads for pure culture or clinical sample, respectively, to obtain a minimum coverage of 1000 per sample. The 300-nucleotide paired-end reads were *de novo* assembled into contigs with the SeqMan NGen software (DNASTAR) using parameters: mer size 31 nucleotides and minimum match percentage 93%. The sizes of resulting contigs produced during analysis of pure cultures ranged from 4183 bp (*Bacillus cereus*) up to 4856 bp (*Acinetobacter parvus*) with an average size of 4423 bp (Table S1). In case of clinical samples, most often an identified bacterial species/genus was represented by a single contig of expected size, around 4.5 kb. However, in some instances only smaller contigs (ranging between 1 and 3 kb in size) represented the same bacterial species/genus present in a sample. In those cases all contigs belonging to the same organism were combined and their reads were added up. Species identification was based on alignment of contig sequences with 16S-23S rRNA sequences deposited in the GenBank database using nucleotide BLAST (Basic Local Alignment Search Tool, http://www.ncbi.nlm.nih.gov/BLAST/). When a reference 16S-23S rRNA sequence was not available in the database, a reference 16S rRNA gene sequence was used for a species identification. The contig sequences were submitted via the website and bacterial species was assigned when the similarity score was 99% or higher and the similarity score differences of the first match with the next closest species was equal to or greater than 0.2%. This reflected a 9 or more nucleotides difference for a sequence of 4423 bp (the average size of the 16S-23S rRNA amplicon). When the similarity score was between 90% and 99%, the genus could be assigned. The score below 90% was interpreted as an unidentified organism.

### Statistical analysis

The four bacterial identification methods were compared using McNemar’s test, a test of paired proportions. Only a single strain per species was taken (from Table S1) for calculation of the accuracy of bacterial identification at the species or genus level according to McNemar’s test. When the *P* value was less than 0.05, we concluded that there was a significant difference between the methods. Statistical analysis was performed using the GraphPad software.

## Electronic supplementary material

Dataset 1
